# Dilated perivascular spaces can present incidental CSF-isointense foci within the ventral forebrain of dogs and cats in transverse MR images

**DOI:** 10.3389/fvets.2022.1002836

**Published:** 2022-10-10

**Authors:** Carolin Fischer, Sebastian Schaub, Kathrin Büttner, Katinka Hartmann, Martin Jürgen Schmidt

**Affiliations:** ^1^Department of Veterinary Clinical Sciences, Clinic for Small Animals, Surgery, Justus-Liebig-University Giessen, Giessen, Germany; ^2^Department for Biomathematics and Data Processing, Justus-Liebig-University Giessen, Giessen, Germany; ^3^VETCARE, Pferde- und Kleintierpraxis AG, Therwil, Switzerland; ^4^Department of Veterinary Clinical Sciences, Clinic for Small Animals, Neurosurgery, Neuroradiology and Clinical Neurology, Justus-Liebig-University Giessen, Giessen, Germany

**Keywords:** (dilated) Virchow-Robin-Space, dog, cat, brain, MRI, CSF, perivascular

## Abstract

**Objective:**

Virchow-Robin-Spaces (VRS) are cerebrospinal fluid (CSF)-containing perivascular spaces encompassing brain vessels while coursing through the parenchyma. VRS can enlarge and become visible in magnetic resonance imaging (MRI). While dilatated VRS are mostly incidental findings, they were associated with degenerative brain disease in humans. This study aimed to evaluate their occurrence and MRI morphology within the ventral forebrain of structurally normal canine and feline brains and physiological cerebrospinal fluid analysis.

**Sample:**

Retro- and prospective, observational study reviewing medical records of client-owned dogs and cats which underwent MRI brain scans for unrelated reasons between 2011 and 2021. We comprised studies with various magnetic field strengths (1 Tesla/3 Tesla). Out of 2500 brain scans, three hundred thirty-five patients (293 dogs, 42 cats) presented with absent intracranial pathology and physiological CSF analysis and were included.

**Procedure:**

The ventral forebrain of the included animals was assessed for bi- or unilateral CSF-isointense foci in the transverse plane. Statistical correlations were evaluated between dilated VRS presence, field strength, age, gender, weight, and cranium conformation. Additionally, a post-mortem histopathologic analysis of one dog and one cat showing dilated VRS on MRI was performed to confirm perforating arteries in the gray matter of the ventral forebrain.

**Results:**

57% of patients presented dilated VRS (*N* = 191: 170 dogs, 21 cats). 43% did not display dilated VRS (control group; *N* = 144: 123 dogs, 21 cats). A significant relation between increased magnetic field strength and detection of dilated VRS was observed in dogs; there was a 2.4 increase (*p* = 0.0001) in detection using 3 Tesla vs. 1 Tesla. There was a 2.4-fold increase in dilated VRS occurrence in male dogs compared to female dogs. Detection also increased with the rise of body weight. We detected no statistically significant difference between dilated VRS and the control group in age, species or cranium conformation.

**Conclusion and Clinical Relevance:**

Dilated VRS can be seen within the ventral forebrain at the level of the rostral commissure on transverse MR images as symmetrical or unilateral, dot-like, CSF-isointense areas. Understanding their signal intensity features and localization prevents misinterpretation and helps differentiate them from various pathological conditions.

## Introduction

**Figure 1:**
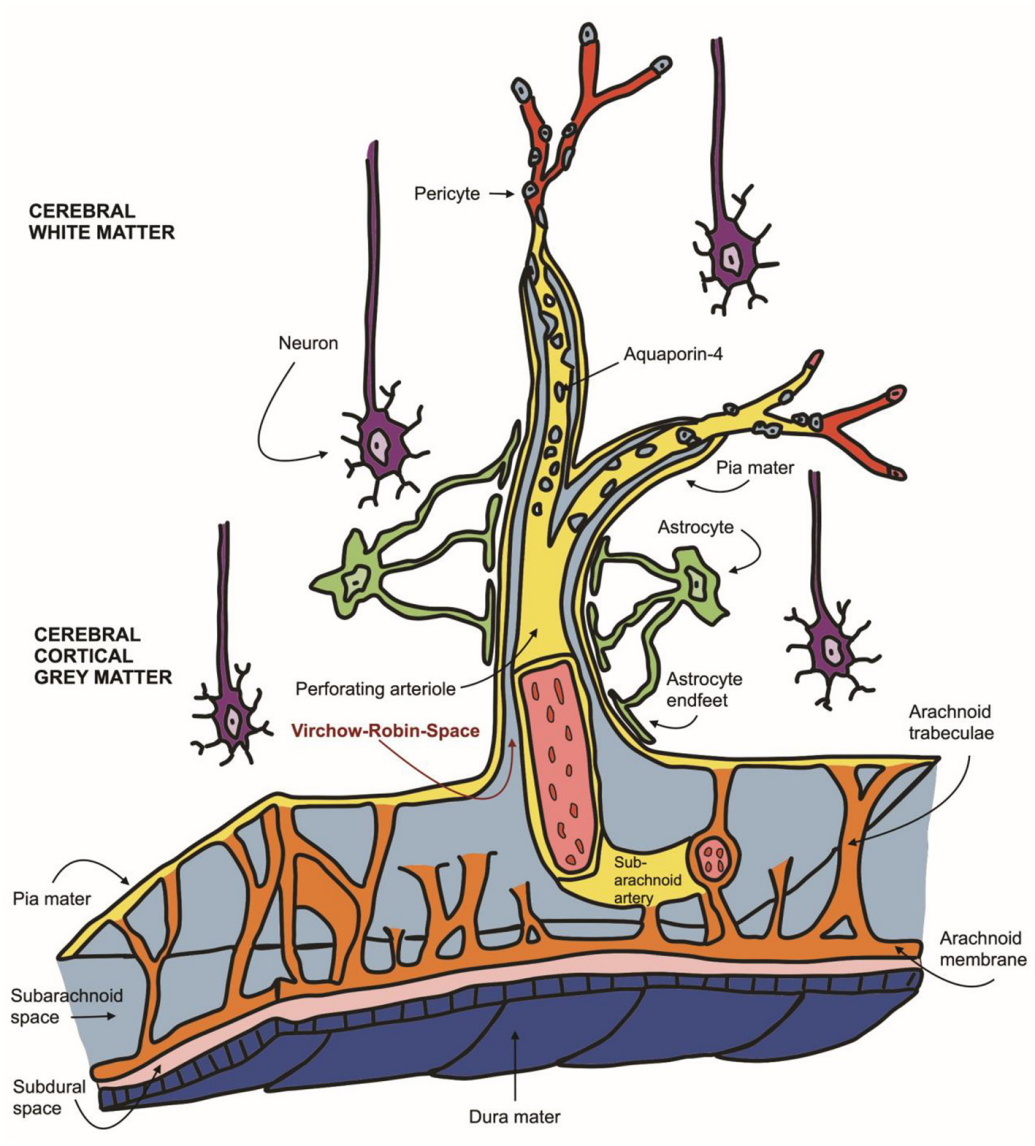
Schematic representation of the neurovascular unit: Virchow-Robin spaces encompassing perforating arteries are filled with CSF as they communicate directly with the subarachnoid space (light blue). VRS (red arrow) are enveloped in two leptomeningeal membranes (yellow), the inner leptomeningeal cell layer facing the arterial wall and the outer membrane, contiguous with the pia mater, facing perivascular astrocytic endfeet (green).

Canine and feline magnetic resonance imaging of the brain is an invaluable, non-invasive diagnostic tool and is considered the gold standard for intraparenchymal changes with a soft tissue contrast superior to computed tomography (1). It allows for the *in vivo* assessment of brain structure, function, maturation and ageing (2, 3). Many intracranial diseases frequently occur in cats and dogs; however, differentiation of subclinical brain lesions from clinically significant ones remains challenging. Neuroanatomical species and breed-associated variations in small animals complicate this further (4). The appearance of perivascular spaces separating blood vessels from the brain parenchyma was first described in detail by the German physician Rudolf Ludwig Carl Virchow in 1851 and confirmed by the French anatomist Charles-Philippe Robin in 1859 (5, 6). They are since known under the eponym Virchow-Robin-Space (VRS). VRS are typically microscopic pial-lined extensions of the subarachnoid space surrounding vessels entering the brain parenchyma (7) see Figure 1. The relevance of these dilated perivascular spaces is not fully understood yet and presents a current field of research. Some authors define them as the lymphatic drainage system of the brain by clearance of the cerebral interstitial fluid derived from blood vessels and grey matter (8). Newer studies describe VRS as part of the glymphatic system, a metabolic waste elimination mainly activated during sleep across all species (9, 10). Several human clinical studies describe expansions of perivascular VRS (10). Widening of VRS often occurs around penetrating arteries and can be seen on transverse MRI slices in all age groups, even in young subjects (7, 10). Their MRI signal intensity is described as isointense to CSF (11). Many studies state they may be physiological and occur independently without an apparent underlying cause (12–19). On the other hand, they are suggested to be associated with the ageing brain (10, 20), present the overture to hypertension (6, 21, 22), white matter hyperintensities (23, 24), small vessel disease (21, 22, 25–29), deposition disorders (e.g., mucopolysaccharidosis) (10, 30), or lacunar infarctions (16, 31, 32).

In a recent review of human medical literature, dilation < 2 mm diameter in the short axis within the basal ganglia, convexity white matter and centrum semiovale and at the mesencephalon were reported as normal findings (33). Significant dilation of the VRS > 3 mm is in humans associated with neurological diseases such as Parkinsonism (34, 35), Alzheimer's disease (2, 8, 36) Multiple Sclerosis, Arteriosclerosis, cerebral amyloid angiopathy (CAA) or small vessel disease (36–39). It is believed, that perivascular enlargement occurs due to impaired metabolic waste that is normally driven by cerebral artery pulsatility. Given the impact of further case management, it is crucial to differentiate dilated VRS from other focal T2 hyperintensities. A judgment on whether dilated VRS in an individual patient is a normal variant or part of a disease process, such as impaired perivascular drainage or perivascular inflammation, can be made by considering the clinical context, the adjacent tissue's appearance on MRI and CSF abnormalities (40). In humans, recognized locations of enlarged perivascular spaces are among others described to be at the inferior basal ganglia (41) or at the proximal part of the lenticulostriate artery, a branch of the middle cerebral artery (31). Furthermore, they are reported to be frequently seen at the level of the rostral commissure (42) (equivalent to the anterior commissure in human medical terminology).

We observed similar small CSF-isointense foci on either side of the rostral commissure in the ventral forebrain of many dog and cat brains within a wide age range. Even though dilated VRS are considerably often reported in humans and laboratory rodents (12–14, 18), to our knowledge, their occurrence and MRI attributes in dogs and cats have not been described other than a single canine report (43). Given the controversies described in the literature, we conducted a retro- and prospective study investigating the significance and MRI morphology of dilated VRS in dog and cat brains. The study aimed to determine the occurrence of dilated perivascular spaces within the ventral forebrain of otherwise structurally normal dog and cat brains. We hypothesized that the CSF-isointense foci within this area present common physiologic variants in the form of dilated Virchow-Robin-Spaces. Secondly, we hypothesized that their occurrence would be similar between dogs and cats and easier to detect using a higher magnetic field strength (3 Tesla vs. 1 Tesla). We believe an understanding and knowledge of their occurrence will help determine whether the presence of these findings should raise the neurologist's or reporting radiologist's concern.

## Materials and methods

### Animals

In this single-centre, descriptive, retro- and prospective observational study, medical records of canine and feline patients that underwent a brain MRI scan performed between November 2011 and November 2021 were reviewed and considered for inclusion. MRI scans were performed for varying purposes unrelated to this study. Clients provided informed consent to use patient data and institutional animal care at hospital admission. The use of ethics committee approval was not necessary. Patients were eligible for inclusion when a complete MRI examination of the brain was performed, including at a minimum: T2-weighted (T2-W) sagittal, dorsal and transverse plane, transverse fluid-attenuated inversion recovery (FLAIR), transverse susceptibility-weighted-image (SWI) (3 Tesla) or T2^*^ (1 Tesla) and T1-weighted (T1-W) pre-contrast sequences. T1-W post-contrast sequences were included, if available. Dogs and cats had to be diagnosed with structurally normal brains, and CSF analysis, performed within 24 h of the MRI study, had to be physiological. For each CSF sample, total nucleated cell count (TNCC), total protein (TP) and cytological evaluation were recorded. Physiological CSF values were considered with a TNCC ≤ 5 cells/μl and TP ≤ 0.3 g/L. For definition of CSF blood contamination, a 500 RBCs/μl cut-off value was used and such samples were excluded from the study (44). Animals were excluded if MRI sequences were incomplete, were of low diagnostic quality, displayed intracranial pathologies such as brain atrophy, anomalies, vascular, infectious, degenerative, neoplastic, traumatic or metabolic changes or if CSF abnormalities were detected. Dogs and cats were consecutively enrolled, provided they met no exclusion criteria. Out of more than 2,500 reviewed brain MRI scans, 335 client-owned animals (293 dogs and 42 cats) were enrolled. Patients were of various breeds, including dolichocephalic, mesocephalic and brachycephalic cranium conformations and various weight classes (<10, 10–30, >30 kg). Enrolment decisions were made by consensus of three study investigators a radiology resident, a board-certified radiologist, and a board-certified neurologist. If dogs met inclusion criteria more than once, only data from the initial examination was used. Patient data collected from the medical records contained signalment, including clinical history, presenting complaints, age at which MRI study was conducted, weight, cranium morphology and CSF analysis results. At initial presentation, all patients were examined and their neurological status recorded by a board-certified veterinary neurologist or veterinary neurology resident-in-training under supervision. The reason for the performed MRI study included one or more of the following symptoms: epileptic seizure, peripheral vestibular syndrome, facial nerve paralysis or blindness. Subsequent to physiological brain MRI scans and normal CSF results, the final clinical diagnosis was other than structural or inflammatory intracranial disease (i.e., primary epilepsy, idiopathic facial nerve paralysis, idiopathic vestibular syndrome, otitis media or sudden acquired retinal degeneration). In some animals, clinically showing behavioural changes such as compulsive disorders or aggression, a definitive diagnosis could not be achieved.

### MRI scan

Our hospital's diagnostic imaging department acquired all MRI scans. Scans obtained between 2011 and 2016 were acquired with a 1 Tesla field strength magnet (Philips Gyroscan Intera). From 2016 to 2021, studies were performed with a 3 Tesla scanner (MAGNETOM Verio 3 Tesla, Siemens Healthcare GmbH). Images were obtained using a loop coil for <5kg patients and a medium flex coil for medium and large patients. A standard clinical conventional MRI protocol was conducted. The protocol included at least the following sequences: T2-W dorsal, transverse and sagittal views, transverse FLAIR, transverse SWI (3 Tesla) or T2^*^ (1 Tesla), dorsal Diffusion-weighted, as well as a T1-W 3D sequence pre and post IV administration of a gadolinium-chelated contrast agent (Omniscan^®^). Depending clinical indication and included neuroanatomical localisations, MRI acquisition time ranged from 30 to 60 min. 1 Tesla images were acquired using the following sequence parameters: T2-W: sequence type 2D; field of view range 18–22 cm; acquisition matrix range 288 × 196 – 352 × 294; slice thickness 3.0–4.0 mm; TR (time of repetition) 4,000–5,500 ms; TE (time of echo) 85–100 ms; flip angle 90; number of averages 3–6. FLAIR: sequence type 2D; field of view range 17–21 cm; acquisition matrix range 288 × 212–336 × 202; slice thickness 3.0–4.0 mm; TR 7,000–8,000 ms; TE 100–120 ms; TI (time of inversion) 2,000–2,400; flip angle 90; number of averages 2–4. T1-W: sequence type 3D; field of view range 14–21 cm; acquisition matrix range 240 × 214–416 × 358; slice thickness 2.0–3.0 mm; TR 300–500 ms; TE 7–16 ms; flip angle 30–90; number of averages 3. DIFF: sequence type 2D; field of view range 13–15 cm; acquisition matrix range 122 × 71–288 × 212; slice thickness 5.0 mm; TR 2,300–2,500 ms; TE 130–150 ms; flip angle 90; number of averages 3. T2^*^-W: sequence type 2D; field of view range 15–18 cm; acquisition matrix range 256 × 192–256 × 256; slice thickness 3.0–4.0 mm; TR 800–1,200 ms; TE 20–25 ms; flip angle 20; number of averages 1. 3 Tesla images were acquired using the following sequence parameters: T2-W: sequence type 2D; field of view range 9–14 cm; acquisition matrix range 256 × 192–320 × 240; slice thickness 3.0 mm; TR 4,000–5,500 ms; TE 75–120 ms; flip angle 90–130; number of averages 4. FLAIR: sequence type 2D; field of view range 12–18 cm; acquisition matrix range 256 × 179–256 × 192; slice thickness 3.0 mm; TR 300–600 ms; TE 3–9 ms; TI 2,000–2,200; flip angle 150; number of averages 2. T1-W: sequence type 3D; field of view range 12 16 cm; acquisition matrix range 128 × 118–256 × 256; slice thickness 1.2 mm; TR 300–600 ms; TE 9–9 ms; flip angle 150; number of averages 2. DIFF: sequence type 2D; field of view range 12–16 cm; acquisition matrix range 120 × 76–120 × 120; slice thickness 3.0 mm; TR 4,000–4,100 ms; TE 64 ms; flip angle 180; number of averages 1–3. SWI: sequence type 3D; field of view range 19–22 cm; acquisition matrix 269 × 384; slice thickness 1.2 mm; TR 28 ms; TE 20 ms; flip angle 15; number of averages 1. All patients were anaesthetized. Anaesthetic protocols were individually tailored to the patient by the attending anaesthetist. Following endotracheal intubation, anaesthesia was maintained with isoflurane (1–3 Vol% CP Pharma, GmbH Germany), oxygen and room air. During anaesthesia, the parameters monitored included heart rate, oxygen saturation, and end-tidal carbon dioxide. Patients were in standardized dorsal recumbency with flexed front limbs fixated over the thorax.

### Image review

**Figure 2:**
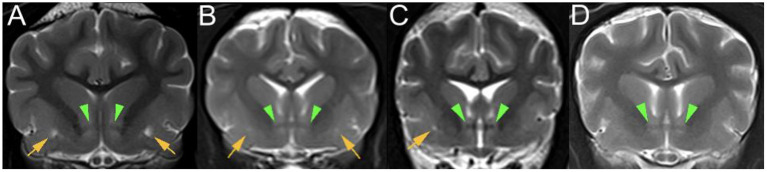
T2-W (3 Tesla) transverse plane images of various dog brains at the level of the rostral commissure (green arrowheads) presenting: **(A)** bilaterally symmetrical, strong focal T2 hyperintensities (yellow arrows) within the ventral forebrain; **(B)** bilaterally symmetrical, faint focal T2 hyperintensities (yellow arrows) within the ventral forebrain; **(C)** unilateral right-sided, strong focal T2 hyperintensity (yellow arrow) within the ventral forebrain; **(D)** control group without CSF-isointense foci in the ventral forebrain at either side of the rostral commissure.

**Figure 3:**
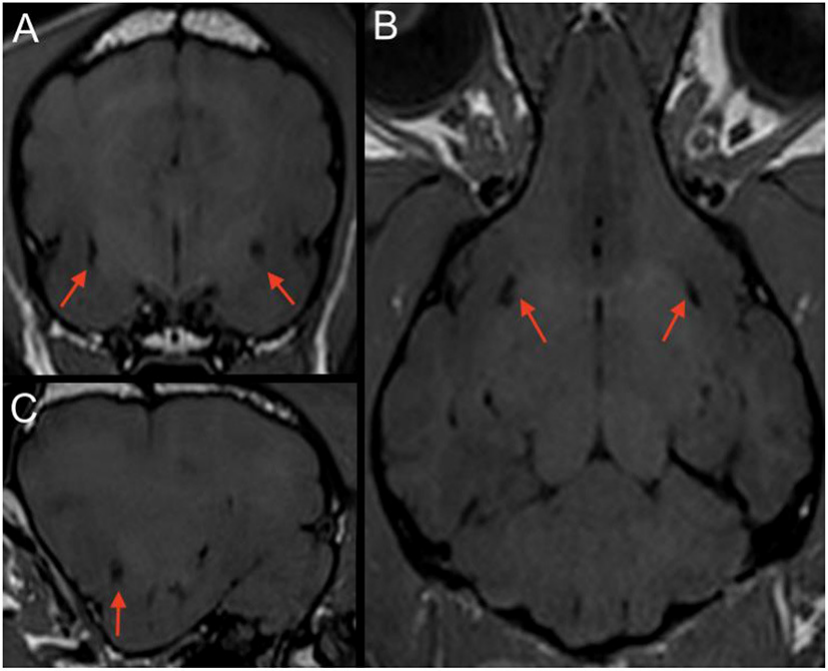
T1-W (3 Tesla) multiplanar reconstruction of a dog with bilateral CSF-isointense foci in the ventral forebrain (red arrows) presenting dilated VRS. Note a relatively round appearance when scanned perpendicular to the imaging plane in the transverse view **(A)**; **(B)** corresponding T1-W dorsal plane, demonstrating their linear presence when running along the imaging plane. **(C)** corresponding parasagittal image.

MRI images were retrieved from the PACS (Picture Archiving Communication System) and reviewed by the first author (ECVDI resident) under the supervision of a board-certified radiologist and neurologist to reach a consensus. Observers were blinded to medical record findings during MRI image evaluation. Magnetic resonance images were viewed using an open-source imaging viewer (HorosTM, The Horos project, Purvis, Annapolis, Maryland, USA). Images were reviewed in all available sequences and evaluated for bilateral symmetrical or unilateral well-delineated, focal, CSF-isointense (T2-W hyper-, FLAIR and T1-W hypointense) areas in a predefined region of interest. The region of interest was at either side of the rostral commissure within the ventral forebrain's grey matter, presenting the territory of the lenticulostriate artery, which enters *via* the rostral perforated substance ventrally. The presence of dilated VRS within the ROI was best identified in T2-W transverse planes, where dilated VRS had a dot-like, non-continuous shape, see Figure 2. In dorsal and sagittal planes, they were identified as fine, linear rostroventral and lateral coursing structures continuous across multiple consecutive slices, see Figure 3. The presence or absence of dilated VRS within the predefined region was recorded. If present, their distribution (bilateral or unilateral) within each cerebral hemisphere was further recorded. Signal intensity was subjectively reported (strong or faint). Furthermore, T1-W pre-and post-contrast images were reviewed for contrast enhancement of the dilated VRS. SWI (3 Tesla) and T2^*^ (1 Tesla) sequences were reviewed, if available, to rule out microbleeds and Diffusion-weighted images to rule out restricted diffusion within the region of interest. Representative T2-W transverse plane images exemplifying the presence of dilated VRS (including bilateral symmetric strong and faint signal intensity and unilateral distribution), and dilated VRS absence (control group) are shown in Figure 2. Representative dorsal plane T1-W slices showing the continuous linear CSF-isointense coursing of the dilated VRS, as well as an exemplary diffusion-weighted apparent diffusion coefficient (ADC) map (Supplementary Figure 1), can be found online in the Supplementary material of this article.

### Histopathological analysis

**Figure 4:**
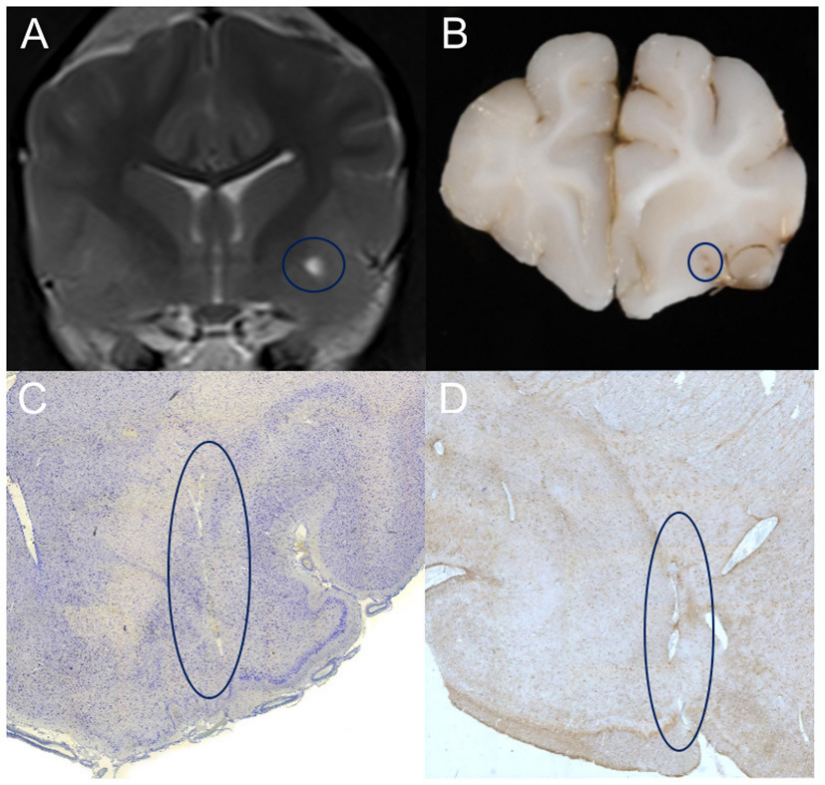
**(A)** T2-weighted transverse MR image of a patient showing left-sided, unilateral dilated VRS on 3 Tesla MRI. Note the highlighted region (blue circle) within the grey matter of the ventral forebrain, the territory of the lenticulostriate arterioles. Corresponding macroscopic **(B)** and microscopic **(C, D)** appearance of the same area of interest in Iba.1 **(C)** GFAP **(D)** stain; note the regular round border of the dilated perivascular spaces enclosing the perforating artery in the centre and the surrounding gliotic nervous tissue.

Post mortem histopathologic analysis of one dog and one cat of the VRS group, displaying unilateral T2 hyper-intensity on MRI, euthanized for reasons unrelated to this study were performed. The brains were harvested within 24 h following euthanasia and stored in 10% neutral buffered formalin for at least 10 days. The fixed neurological tissue was trimmed in 4 mm slices within the predefined region of interest and embedded in paraffin. Blocks were sectioned in histopathological slices using a microtome and stained with Factor VIII, GFAP (glial fibrillary acidic protein), Iba.1 (ionized calcium-binding adapter molecule 1 antibody) and Luxol-Kresyl. A board-certified veterinary pathologist confirmed the normal status of the tissue, the presence of ventrally perforating arteries within the ventral forebrain and astroglial cells surrounding these perforating vessels in GFAP and Iba.1 stain, see Figure 4.

### Statistical analysis

Statistical analysis was performed by the head of the biomathematics and data processing department using commercial software (SAS^®^ Institute Inc., 2013. Base SAS^®^ 9.4 Procedures Guide: Statistical Procedures, 2nd edition ed. Statistical Analysis System Institute Inc., Cary, NC, USA). A chi-square test of independence and Fisher's exact test were performed on categorical variables to determine significant relationships between each variable. Categorial variables included the appearance of the finding and magnetic field strength (1 Tesla vs. 3 Tesla). Logistic regression analyses were performed to quantify the strength of association between the presence or absence of the finding within the investigated species (dog vs. cat), age when undergoing MRI examination, sex, weight group (<10, 10–30, >30 kg). A chi-square test was also used to test for any significant difference when comparing cranium morphology between groups (brachycephalic, dolichocephalic, mesocephalic). Unadjusted *P*-values <0.05 were considered statistically significant.

## Results

**Figure 5:**
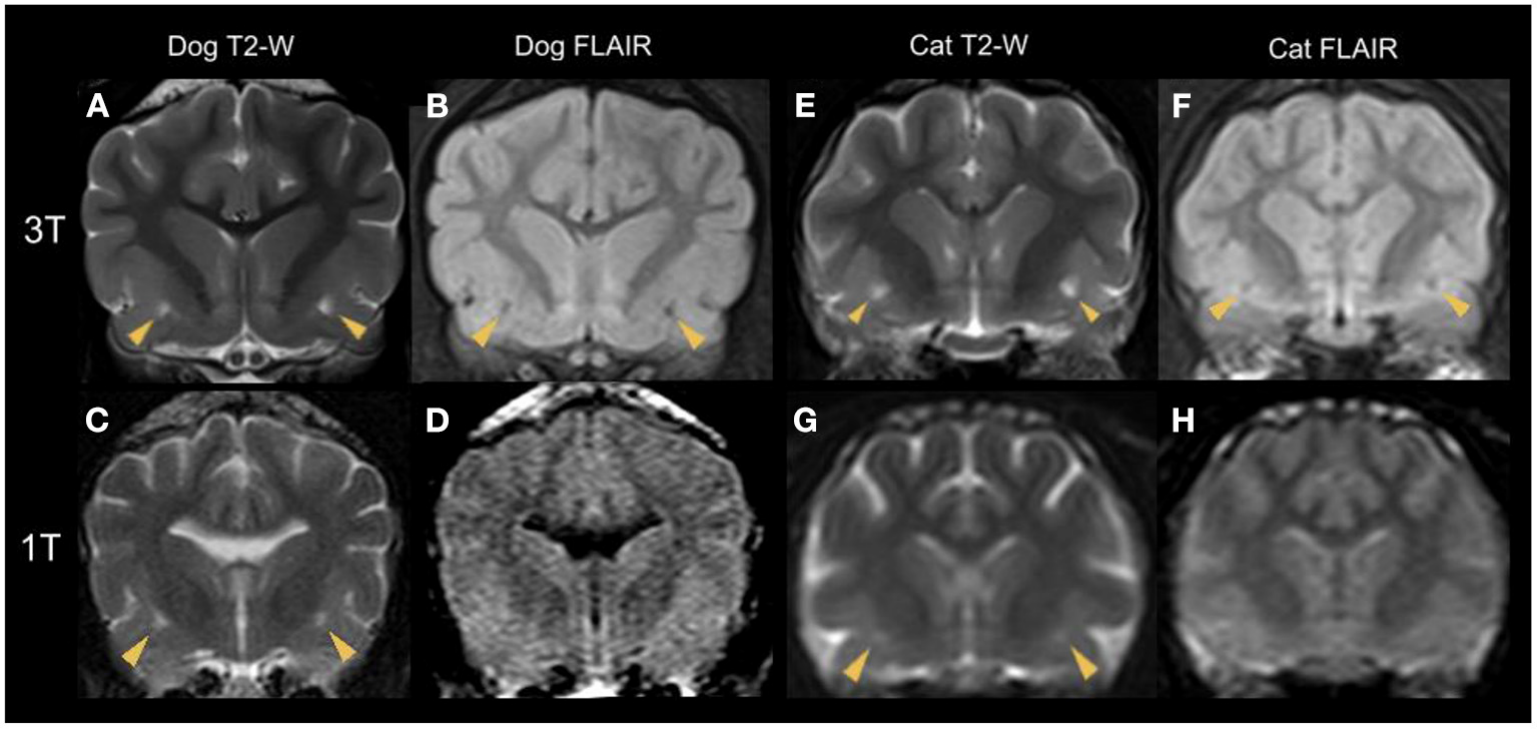
Transverse plane MR images of various dog **(A–D)** and cat **(E–H)** brains at the level of the rostral commissure with bilateral dilated VRS (yellow arrowheads). T2-W **(A,C,E,G)** and corresponding FLAIR **(B,D,F,H)** sequences are shown. The top line **(A,B,E,F)** demonstrates sequences acquired with a 3 Tesla scanner and the bottom line **(C,D,G,H)** presents 1 Tesla studies. Note the hypointense signal intensity in FLAIR, confirming CSF-isointensity. Due to the decreased image resolution of the 1 Tesla FLAIR studies, the CSF-isointense foci cannot be detected **(D,H)**.

A total of three hundred thirty-five (335) animals were eligible for enrolment based on the inclusion criteria. The study population consisted of 293 dogs and 42 cats, of which 35 brachycephalic animals were included (34 dogs, one cat). 57% (*N* = 191) of the animals were included in the dilated VRS group (170 dogs, 21 cats). 43% (*N* = 144) of the dogs and cats displayed no dilated VRS and formed the control (123 dogs, 21 cats), see Table 1. At the time of MRI examination, animals of the dilated VRS group had a mean age of 5 years (dogs 3 months−15 years; cats 6 months−15 years). Dogs and cats of the control group had a mean age of 5, 6 years (dogs 5 months−15 years; cats 6 months−16 years). When both groups' ages and species were compared, no statistical significance was detected between the dilated VRS group and the control group. Age distribution was nearly normal in dogs and normally distributed in cats. There was no statistically significant difference between the occurrence of dilated VRS and species. There was a statistically significant difference between field strength and dilated VRS detection within the canine patient pool. 69.11% of the dilated VRS group underwent 3 Tesla MRI scans, whereas only 48.25% of the control group animals were examined using 3 Tesla MRI. Dilated VRS were 2.4 times more conspicuous with 3 Tesla acquired sequences compared to 1 Tesla acquired sequences (*p* = 0.0001), see Figure 5. There was no statistical difference in the occurrence of dilated VRS and field strength in cats. There was a statistically significant correlation between sex and dilated VRS occurrence; 68.59% of dilated VRS animals were male (*p* = 0.0007). Within the entire patient pool, there was a 2.5 fold increase (OR) of male patients presenting with dilated VRS. In the group of dogs showing dilated VRS, 69.82% were male (2.43 OR). There was no statistically significant difference in dilated VRS detection in male entire vs. male neutered canine patients. The patient weight distribution was nearly normal in dogs and was normally distributed in cats. Cats did not confirm a statistical difference in the occurrence of dilated VRS and sex. A significant difference in weight between dogs showing dilated VRS and dogs of the control group was noted. Detection of dilated VRS in dogs weighing 10–30 kg was 2.5 fold increased compared to animals weighing <10 kg (*p* = 0.0014), and there was a 3.0-fold increase (*p* = 0.0091) of dilated VRS in dogs weighing >30 kg compared to animals with a weight of <10 kg. A significant difference in detection between weight class 2 (10–30 kg) and weight class 3 (>30 kg) was not detected. Statistical difference in the occurrence of dilated VRS and weight was not confirmed in cats. Furthermore, no difference was noted when comparing the weight of cats and small dogs (>10 kg) with dilated VRS. There was no statistical association between cranium conformation and dilated VRS occurrence between both groups dogs *p* = 0.1704, cats *p* > 0.05). However, brachycephalic dogs were underrepresented in both groups. Due to the smaller feline sample size, an exact Pearson Chi-square test further confirmed no statistical association between cranium conformation and dilated VRS occurrence (*p* > 0.05). In 16 patients (8%) with dilated VRS and 13 patients (9%) of the control group a final diagnosis could not be reached, those patients showed clinically behavioural changes such as compulsive disorders or aggression.

**Table 1:** Demonstrating the distribution of patient criteria subdivided into both study groups (dilated VRS and control group).

	**335 patients (293 dogs, 42 cats)**					
	**Dilated VRS group**			**Control group**		
**Species**	**Dogs**	**Cats**	**Both**	**Dogs**	**Cats**	**Both**
	170	21	191	123	21	144
**Field Strength**						
1 Tesla *N =* 133	52	7	59	64	10	74
3 Tesla *N =* 202	118	114	132	59	11	70
**Weight class**						
<10 kg	29	13	42	45	11	56
10–30 kg	97	1	98	61	0	61
>30 kg	49	0	49	27	0	27
**Sex**						
Male	118	13	131 (43,5% neutered)	60	12	72 (50% neutered)
Female	52	8	60 (65% neutered)	63	9	72 (63,8% neutered)
**Age MRI performed**	**5 years**			**5.6 years**		
**Cranium morphology**						
Dolicho-/mesocephalic	156	20	176	101	21	122
Brachycephalic	14	1	15	20	0	20

## Discussion

Based on our literature review, the present investigation is the first to assess dilated VRS occurrence within a large sample size of dogs and cats as a normal finding. In line with human studies (7, 8, 12–15, 17–19) the results support our hypothesis that these CSF-isointense foci at either side of the rostral commissure, best seen on transverse plane T2-W MR images, represent common normal imaging findings. Histopathologic examinations confirmed our first hypothesis, as penetrating arteries (lenticulostriate arteries, branching off the middle cerebral artery) are seen bilaterally within the ventral forebrain. The present work's strengths include using a relatively large, heterogeneous study population, including 335 patients of two different species and various breeds, a wide age range, and the inclusion of different magnetic field strengths. The stability of the characteristic MRI features combined with the post-mortem histopathology results increased our diagnostic confidence that the described foci demonstrate cross-sections of dilated Virchow-Robin-Spaces, equivalent to human neuroimaging findings. A judgement on whether a dilated VRS in an individual patient is a normal variant or part of a disease process can be made by considering the adjacent tissue's appearance on MRI and the clinical context (40). None of our patients displayed any adjacent tissue abnormalities, such as diffuse T2 and FLAIR hyperintensities surrounding the described dilated VRS.

In human geriatric medicine, enlarged perivascular spaces are associated with progressive degenerative brain diseases such as Parkinsonism (34, 35), Alzheimer's (2, 8, 36), Multiple Sclerosis, Arteriosclerosis, cerebral amyloid angiopathy (CAA) or small vessel disease (36–39). However, we did not find any statistical association with patient age in the current study. Due to the wide age distribution of included animals, a congenital aetiology is supported by the dilated VRS in a dog as young as 3 months and a cat as young as 6 months. Given the occurrence within all age groups and breeds, dilated VRS could also reflect changes occurring at varying stages of development or genetic variability. As brains age and the brain parenchyma atrophies, dilated VRS can become more apparent (22, 26, 36, 45), potentially playing a role in our geriatric dilated VRS group patients. However, the eldest patient in the study group, a 16-year-old dog, displayed no dilated VRS. Furthermore, animals with objectively significant brain atrophy were excluded from the study. Future studies investigating an increased prevalence of dilated VRS in dogs and cats diagnosed with brain atrophy would be interesting. Analysing an association between dilated VRS occurrence and loss of cognitive function of the included animals, as described in human dementia patients (45) was beyond the scope of the present study. Moreover, cognitive dysfunction is difficult to evaluate in pets and plays a minor role compared to humans.

It is essential to notice a specific limit in MRI resolution of subtle brain structures (40) such as dilated VRS; therefore, we evaluated the ability to detect them by comparing different magnetic field strengths. Based on our statistical analysis, the CSF-isointense foci were 2.4 times more likely to be detected in animals examined with higher magnetic field strength (3 Tesla vs. 1 Tesla). This is not surprising as image resolution and signal-to-noise ratio increase with magnetic field strength incline, enabling excellent detail detection (46, 47); for comparison, see Figure 3. We furthermore noticed a less evident FLAIR hypointense signal intensity of the described dilated VRS in 1 Tesla acquired FLAIR images compared to 3 Tesla FLAIR sequences, see Figure 5. A recent human medicine study offers detailed 3D visualisation of perivascular spaces using 7 Tesla (47). Our study also provides evidence that dilated VRS are visible with 1 Tesla systems in dogs and cats; however, 1 Tesla scanners are less sensitive than those using higher magnetic field strengths (48). Comparing dilated VRS detection of one patient examined in both included magnetic field strengths would have enriched our study but was impossible due to the retrospective study design. Furthermore, it would be interesting to examine dilated VRS occurrence and detectability in low-field MRI studies, given that they are still broadly used in veterinary medicine. If dilated VRS are large enough, we postulate they might be seen at least in the 3D sequences obtained with those scanners.

We found no statistically significant disparity in signal occurrence within our mixed-species population. Reports of human medicine, studies of laboratory rodents (12–15, 18), and previous canine reports (2, 43) equally support the interspecies occurrence of dilated VRS. We believe the occasional unilateral occurrence, faint signal intensity or absence of the examined dilated VRS in the T2-weighed transverse MR images can be explained by a relatively large slice thickness of 2–4 mm regarding the small short-axis diameter of the examined structures. Alternatively, they present a normal variant of the respective patient. Especially in small patients, relatively large slice thicknesses or mild oblique angulation during image acquisition could have precluded our ability to detect dilated VRS within the ventral forebrain.

The weight class played a significant role in the signal presence statistically; there was a 2.5-fold increase in dilated VRS occurrence within weight class 2 (10–30 kg) and a three-fold increase in weight class 3 (>30 kg). In cats, we did not confirm this finding, likely due to the homogenous weight distribution of this species (99% <10 kg). There was also no statistically significant difference in dilated VRS occurrence comparing cats with small dogs weighing <10 kg. As voxel size is adjusted based on the patient head size (small patients: pixel spacing 0.2 × 0.2 mm, medium patients: pixel spacing 0.31 × 0.31 mm, large patients: pixel spacing 0.43 × 0.43 mm), we believe that the higher dilated VRS incidence with increased patient weight could be proportional to dilated VRS diameter. However, this is an assumption; the impact of weight needs to be interpreted with caution.

Comparable to results from human studies (22, 36, 45), we found a statistically significant increased occurrence of dilated VRS in male dogs, independent of neuter status. This finding remains unexplained in humans as well as in veterinary medicine. There was no significant association in feline gender, likely due to the smaller sample size of included cats. Care should be taken not to confuse our findings with white matter or small vessel disease, which prompt careful screening for risk factors of stroke and dementia in humans (24). White matter lesions are present if hyperintense on T2-W, FLAIR, and proton density images, without prominent hypointensity on T1-W images (24). In this study, we are not referring to white matter lesions, as our described T2-W hyperintensities present hypointense in FLAIR sequences and occur in the gray matter of the ventral forebrain rather than white matter areas. Moreover, cerebral white matter lesions often occur in cortical regions (6, 47) rather than subcortical grey matter of the ventral forebrain, as described in our study.

When comparing post-mortem gross or histopathology samples with MRI images, changes resulting from fixation, like shrinkage, must be considered (25). After cutting 1-2 cm transverse sections, only taking macroscopically interesting regions, the correct slice corresponding to the transverse MRI image could have been missed. Nevertheless, lacunar strokes in human medicine are often found within the territory of the lenticulostriate arteries and usually present perivascular edema and thickening, inflammation and disintegration of the arteriolar wall (49); such changes were not found in the histopathological analysis of our two included patients. Furthermore, a previous MRI study claims lacunar infarcts appear rather wedge-shaped in humans (31). Likewise, they are triangular in dogs and cats (50) rather than uniformly round as the dilated VRS we describe. Subsequent studies evaluating an association of dilated VRS detection in brains with concurrent brain disease (e.g., neoplasia, inflammatory changes, haemorrhage) are required to expand our findings and improve the overall diagnostic yield.

Interestingly, the results of this study did not support any significant correlation between cranium conformation and dilated VRS occurrence. However, within the study group, brachycephalic breeds were too underrepresented compared to dolichocephalic and mesocephalic breeds to conclude this precisely. We had a moderate selection bias for animals with epileptic seizures, as these are patients most likely presenting for MRI evaluation of the brain. Primary epilepsy is defined by structurally normal brain parenchyma and physiologic CSF analysis (51); therefore, epileptic dogs and cats meeting these criteria were not excluded from the study.

Inherent to the retrospective nature of the data analysis, the main limitation of this study is the lack of histopathological examination for the majority of the included animals. Definitive microscopic confirmation remains challenging given that the data analysis was obtained in client-owned animals with normal brains and mainly medically treatable conditions, such as primary idiopathic epilepsy. Furthermore, due to our heterogenous study population, we did not investigate the cut-off measurement of the dilated VRS short-axis diameter, similar to human medicine studies (33), although the authors of a human study claim the size of dilated VRS to be the most crucial factor for discrimination from lacunar infarctions; reporting a sensitivity of 86% and specificity of 91% if Virchow-Robin spaces are no larger than 2 × 2 mm (31). Future elucidations potentially obtaining veterinary thresholds in homogenous study populations are warranted. A further weakness of the present study is the limited number of examined cofactors such as hypertension, (inflammatory) blood parameters, cerebral amyloid angiopathy, or use of medications known to affect the cardiovascular system and cerebral perfusion (e.g., sedatives, furosemide, pimobendane, propentofylline or corticosteroids). Including these parameters would have enriched our study and shed light on the relationship between cerebral and systemic conditions and the occurrence of significantly enlarged VRS. However, we did exclude patients with CSF changes in an attempt to account for that. Besides, a previous human medicine study found only a weak association between the rise of inflammatory markers (increased CRP, IL-6) and visible perivascular spaces (48).

In conclusion, this study represents the first step in veterinary medicine investigating dilated perivascular Virchow-Robin-Spaces as CSF-isointense, dot-like hyperintensities in the transverse plane of the ventral forebrain in a large sample of dogs and cats. Our results emphasize improved dilated VRS detection using 3 Tesla vs. 1 Tesla and overrepresentation in male dogs. No significant differences in dilated VRS occurrence between species (dogs and cats), age and cranium conformation were detected. Due to the high prevalence of dilated VRS found in our study (57%), we believe they may be incidental findings in dogs and cats. Care should be taken not to misinterpret these CSF-isointense foci as pathological findings when discovered in the grey matter at either side of the rostral commissure.

## Data availability statement

The raw data supporting the conclusions of this article will be made available by the authors, without undue reservation.

## Ethics statement

Ethical review and approval was not required for the animal study because all MRI scans and CSF analyses were performed for reasons unrelated to this study. Written informed consent for participation was not obtained from the owners because MRI scans and CSF analyses were obtained for reasons unrelated to this study.

## Author contributions

CF: study design, data analysis, and generation of the manuscript. MS and SS: study design, data analysis, and correction of the manuscript. KH: Generation of the manuscript. KB: Statistical analysis. All authors contributed to the article and approved the submitted version.

## Conflict of interest

Author KH was employed by company VETCARE, Pferde- und Kleintierpraxis AG. The remaining authors declare that the research was conducted in the absence of any commercial or financial relationships that could be construed as a potential conflict of interest.

## Publisher's note

All claims expressed in this article are solely those of the authors and do not necessarily represent those of their affiliated organizations, or those of the publisher, the editors and the reviewers. Any product that may be evaluated in this article, or claim that may be made by its manufacturer, is not guaranteed or endorsed by the publisher.

## Supplementary material

Supplementary Figure 1 **(A,C)** Transverse plane T2-W image of a canine brain with bilateral CSF-isointense foci in the ventral forebrain at the level of the rostral commissure. **(B,D)** corresponding dorsal plane Diffusion-Weighted ADC map showing no sign of restricted diffusion at the same location, indicated by the crosshairs.

Supplementary Video Representative dorsal plane T1-W slices showing the continuous linear CSF-isointense coursing of the dilated Virchow-Robin-Spaces (VRS).

## References

[1] Thomson CE DVM JNK Burn RA Drayer BP Hadley DM Levesque DC Gainsburg LA DVM SBL. Magnetic resonance imaging-a general overview of principles and examples in veterinary neurodiagnosis. Vet Radiol Ultrasound. (1993). 10.1111/j.1740-8261.1993.tb01986.x

[2] Cummings BJ Head E Ruehl W Milgram NW Cotman CW. The canine as an animal model of human aging and dementia. Neurobiol Aging. (1996) 17:259–68. 10.1016/0197-4580(95)02060-8 8744407

[3] Courchesne E Chisum HJ Townsend J Cowles A Covington J Egaas B. Normal brain development and aging: quantitative analysis at *in vivo* mr imaging in healthy volunteers1. Radiology. (2000) 216:672–82. 10.1148/radiology.216.3.r00au37672 10966694

[4] Hecht EE Smaers JB Dunn WD Kent M Preuss TM Gutman DA. Significant neuroanatomical variation among domestic dog breeds. J Neurosci. (2019) 39:7748–58. 10.1523/JNEUROSCI.0303-19.2019 31477568 PMC6764193

[5] WOOLLAM DH MILLEN JW. The perivascular spaces of the mammalian central nervous system and their relation to the perineuronal and subarachnoid spaces. J Anat. (1955) 89:193–200. 14367214 PMC1244781

[6] Wardlaw JM Benveniste H Nedergaard M Zlokovic BV Mestre H Lee H. Perivascular spaces in the brain: anatomy, physiology and pathology. Nat Rev Neurol. (2020) 16:137–53. 10.1038/s41582-020-0312-z 32094487

[7] Barkhof F. Enlarged Virchow-Robin spaces: do they matter? J Neurol Neurosurg Psychiatry. (2004) 75:1516. 10.1136/jnnp.2004.044578 15489377 PMC1738815

[8] Bakker ENTP Bacskai BJ Arbel-Ornath M Aldea R Bedussi B Morris AWJ. Lymphatic clearance of the brain: perivascular, paravascular and significance for neurodegenerative diseases. Cell Mol Neurobiol. (2016) 36:181–94. 10.1007/s10571-015-0273-8 26993512 PMC4844641

[9] Jessen NA Munk ASF Lundgaard I Nedergaard M. The glymphatic system: a beginner's guide. Neurochem Res. (2015) 40:2583–99. 10.1007/s11064-015-1581-6 25947369 PMC4636982

[10] Kwee RM Kwee TC. Virchow-Robin Spaces at MR imaging. Radiographics. (2007) 27:1071–86. 10.1148/rg.274065722 17620468

[11] Braffmann. Brain MR: Pathologic Correlation with Gross and Histopathology. AJNR (1988)

[12] Zhang ET Richards HK Kida S Weller RO. Directional and compartmentalised drainage of interstitial fluid and cerebrospinal fluid from the rat brain. Acta Neuropathol. (1992) 83:233–9. 10.1007/BF00296784 1373020

[13] Ichimura T Fraser PA Cserr HF. Distribution of extracellular tracers in perivascular spaces of the rat brain. Brain Res. (1991) 545:103–13. 10.1016/0006-8993(91)91275-6 1713524

[14] Bedussi B Naessens DMP Vos J de Engberink RO Wilhelmus MMM Richard E. ten, vanBavel E, Bakker ENTP. Enhanced interstitial fluid drainage in the hippocampus of spontaneously hypertensive rats. Sci Rep-UK. (2017) 7:744. 10.1038/s41598-017-00861-x 28389645 PMC5429689

[15] Esiri MM Gay D. Immunological and neuropathological significance of the Virchow-Robin space. J Neurol Sci. (1990) 100:3–8. 10.1016/0022-510X(90)90004-7 2089138

[16] Bailey EL McCulloch J Sudlow C Wardlaw JM. Potential animal models of lacunar stroke. Stroke. (2009) 40:e451–8. 10.1161/STROKEAHA.108.528430 19390080

[17] Lee H Mortensen K Sanggaard S Koch P Brunner H Quistorff B. Benveniste H. Quantitative Gd-DOTA uptake from cerebrospinal fluid into rat brain using 3D VFA-SPGR at 94T. Magnet Reson Med. (2018) 79:1568–78. 10.1002/mrm.26779 28627037 PMC5736474

[18] Venkat P Chopp M Zacharek A Cui C Zhang L Li Q. White matter damage and glymphatic dysfunction in a model of vascular dementia in rats with no prior vascular pathologies. Neurobiol Aging. (2017) 50:96–106. 10.1016/j.neurobiolaging.2016.11.002 27940353 PMC5209254

[19] Hirabuki N Fujita N Fujii K Hashimoto T Kozuka T MR. appearance of Virchow-Robin spaces along lenticulostriate arteries: spin-echo and two-dimensional fast low-angle shot imaging. Ajnr Am J Neuroradiol. (1994) 15:277–81. 8192073 PMC8334624

[20] Heier LA Deck MDF. Large Virchow-Robin Spaces: MR-Clinical Correlation. AJNR (1989) 2505536 PMC8335297

[21] Mestre H Kostrikov S Mehta RI Nedergaard M. Perivascular spaces, glymphatic dysfunction, and small vessel disease. Clin Sci. (2017) 131:2257–74. 10.1042/CS20160381 28798076 PMC5567781

[22] Zhu Y-C Tzourio C Soumaré A Mazoyer B Dufouil C Chabriat H. Severity of dilated Virchow-Robin Spaces is associated with age, blood pressure, and mri markers of small vessel disease. Stroke. (2010) 41:2483–90. 10.1161/STROKEAHA.110.591586 20864661

[23] Weidauer S Wagner M Hattingen E. White Matter Lesions in Adults – a Differential Diagnostic Approach. Röfo - Fortschritte Auf Dem Gebiet Der Röntgenstrahlen Und Der Bildgebenden Verfahren. (2020) 192:1154–73. 10.1055/a-1207-1006 32688424

[24] Debette S Markus HS. The clinical importance of white matter hyperintensities on brain magnetic resonance imaging: systematic review and meta-analysis. BMJ. (2010) 341:c3666. 10.1136/bmj.c3666 20660506 PMC2910261

[25] Gouw AA Seewann A Flier WM. van der Barkhof F Rozemuller AM Scheltens P Geurts JJG. Heterogeneity of small vessel disease: a systematic review of MRI and histopathology correlations. J Neurology Neurosurg Psychiatry. (2011) 82:126. 10.1136/jnnp.2009.204685 20935330

[26] Potter GM Doubal FN Jackson CA Chappell FM Sudlow CL Dennis MS. Enlarged perivascular spaces and cerebral small vessel disease. Int J Stroke. (2015) 10:376–81. 10.1111/ijs.12054 23692610 PMC4463944

[27] Bath PM Wardlaw JM. Pharmacological treatment and prevention of cerebral small vessel disease: a review of potential interventions. Int J Stroke. (2015) 10:469–78. 10.1111/ijs.12466 25727737 PMC4832291

[28] Mok V Kim JS. Prevention and management of cerebral small vessel disease. J Stroke. (2015) 17:111–22. 10.5853/jos.2015.17.2.111 26060798 PMC4460330

[29] Brown R Benveniste H Black SE Charpak S Dichgans M Joutel A. Understanding the role of the perivascular space in cerebral small vessel disease. Cardiovasc Res. (2018) 114:1462–73. 10.1093/cvr/cvy113 29726891 PMC6455920

[30] Matheus MG Castillo M Smith JK Armao D Towle D Muenzer J. Brain MRI findings in patients with mucopolysaccharidosis types I and II and mild clinical presentation. Neuroradiology. (2004) 46:666–72. 10.1007/s00234-004-1215-1 15205860

[31] Bokura H Kobayashi S Yamaguchi S. Distinguishing silent lacunar infarction from enlarged Virchow-Robin spaces: a magnetic resonance imaging and pathological study. J Neurol. (1998) 245:116–22. 10.1007/s004150050189 9507419

[32] Jungreis CA Kanal E Hirsch WL Martinez AJ Moossy J. Normal perivascular spaces mimicking lacunar infarction: MR imaging. Radiology. (1988) 169:101–4. 10.1148/radiology.169.1.3420242 3420242

[33] Capasso R Negro A Cirillo S Iovine S Puoti G Cirillo M Conforti R. Large anterior temporal Virchow–Robin spaces: Evaluating MRI features over the years—Our experience and literature review. Clin Transl Neurosci. (2020) 4:2514183X20905252. 10.1177/2514183X20905252

[34] Fénelon G Gray F Wallays C Poirier J Guillard A. Parkinsonism and dilatation of the perivascular spaces (État Criblé) of the striatum: A clinical, magnetic resonance imaging, and pathological study. Movement Disord. (1995) 10:754–60. 10.1002/mds.870100609 8749995

[35] Fernández ML. Virchow-Robin spaces: A cause of parkinsonism? Neurología (2016)

[36] Ramirez J Berezuk C McNeely AA Scott CJM Gao F Black SE. Visible Virchow-Robin Spaces on magnetic resonance imaging of alzheimer's disease patients and normal elderly from the sunnybrook dementia study. J Alzheimer's Dis. (2015) 43:415–24. 10.3233/JAD-132528 25096616

[37] Chen A Akinyemi RO Hase Y Firbank MJ. Ndung'u MN Foster V Craggs LJL Washida K Okamoto Y Thomas AJ. Frontal white matter hyperintensities, clasmatodendrosis and gliovascular abnormalities in ageing and post-stroke dementia. Brain. (2016) 139:242–58. 10.1093/brain/awv328 26667280 PMC4905522

[38] Kalaria RN. Small vessel disease and Alzheimer's dementia: pathological considerations. Cerebrovasc Dis. (2002) 13:48–52. 10.1159/000049150 11901243

[39] Thore CR Anstrom JA Moody DM Challa VR Marion MC Brown WR. Morphometric analysis of arteriolar tortuosity in human cerebral white matter of preterm, young, and aged subjects. J Neuropathology Exp Neurology. (2007) 66:337–45. 10.1097/nen.0b013e3180537147 17483690

[40] Groeschel S Chong WK Surtees R Hanefeld F. Virchow-Robin spaces on magnetic resonance images: normative data, their dilatation, and a review of the literature. Neuroradiology. (2006) 48:745–54. 10.1007/s00234-006-0112-1 16896908

[41] Rawal S Croul SE Willinsky RA Tymianski M Krings T. Subcortical cystic lesions within the anterior superior temporal gyrus: a newly recognized characteristic location for dilated perivascular spaces. Am J Neuroradiol. (2014) 35:317–22. 10.3174/ajnr.A3669 23945225 PMC7965768

[42] Pullicino PM Miller LL Alexandrov AV Ostrow PT. Infraputaminal ‘lacunes’: clinical and pathological correlations. Stroke. (1995) 26:1598–602. 10.1161/01.STR.26.9.1598 7660405

[43] Criswell TP Sharp MM Dobson H Finucane C Weller RO Verma A. The structure of the perivascular compartment in the old canine brain: a case study. Clin Sci. (2017) 131:2737–44. 10.1042/CS20171278 28982724

[44] Doyle C Solano-Gallego L. Cytologic interpretation of canine cerebrospinal fluid samples with low total nucleated cell concentration, with and without blood contamination. Vet Clin Path. (2009) 38:392–6. 10.1111/j.1939-165X.2009.00132.x 19392761

[45] MacLullich AMJ Wardlaw JM Ferguson KJ Starr JM Seckl JR Deary IJ. Enlarged perivascular spaces are associated with cognitive function in healthy elderly men. J Neurology Neurosurg Psychiatry. (2004) 75:1519. 10.1136/jnnp.2003.030858 15489380 PMC1738797

[46] Ma SJ Sarabi MS Yan L Shao X Chen Y Yang Q. Characterization of lenticulostriate arteries with high resolution black-blood T1-weighted turbo spin echo with variable flip angles at 3 and 7 Tesla. Neuroimage. (2019) 199:184–93. 10.1016/j.neuroimage.2019.05.065 31158475 PMC6688958

[47] Bouvy WH Biessels GJ Kuijf HJ Kappelle LJ Luijten PR Zwanenburg JJM. Visualization of perivascular spaces and perforating arteries with 7 T magnetic resonance imaging. Invest Radiol. (2014) 49:307–13. 10.1097/RLI.0000000000000027 24473365

[48] Aribisala BS Wiseman S Morris Z Valdés-Hernández MC Royle NA Maniega SM. Circulating inflammatory markers are associated with magnetic resonance imaging-visible perivascular spaces but not directly with white matter hyperintensities. Stroke. (2014) 45:605–7. 10.1161/STROKEAHA.113.004059 24399375 PMC3906539

[49] Bailey EL Smith C Sudlow CLM Wardlaw JM. Pathology of lacunar ischemic stroke in humans—a systematic review. Brain Pathol. (2012) 22:583–91. 10.1111/j.1750-3639.2012.00575.x 22329603 PMC8057646

[50] Mai W. Diagnostic MRI in dogs and cats. (2018) 10.1201/9781315121055

[51] Koestner A. Neuropathology of canine epilepsy. Problems Vet Medicine. (1989) 1:516–34. 2520132

