# Supplementary figures and images for: Dilated perivascular spaces can present incidental CSF-isointense foci within the ventral forebrain of dogs and cats in transverse MR images
Source: Front Vet Sci. 2022 Oct 10;9:1002836. doi: 10.3389/fvets.2022.1002836 (PMC9590410; doi:10.3389/fvets.2022.1002836)

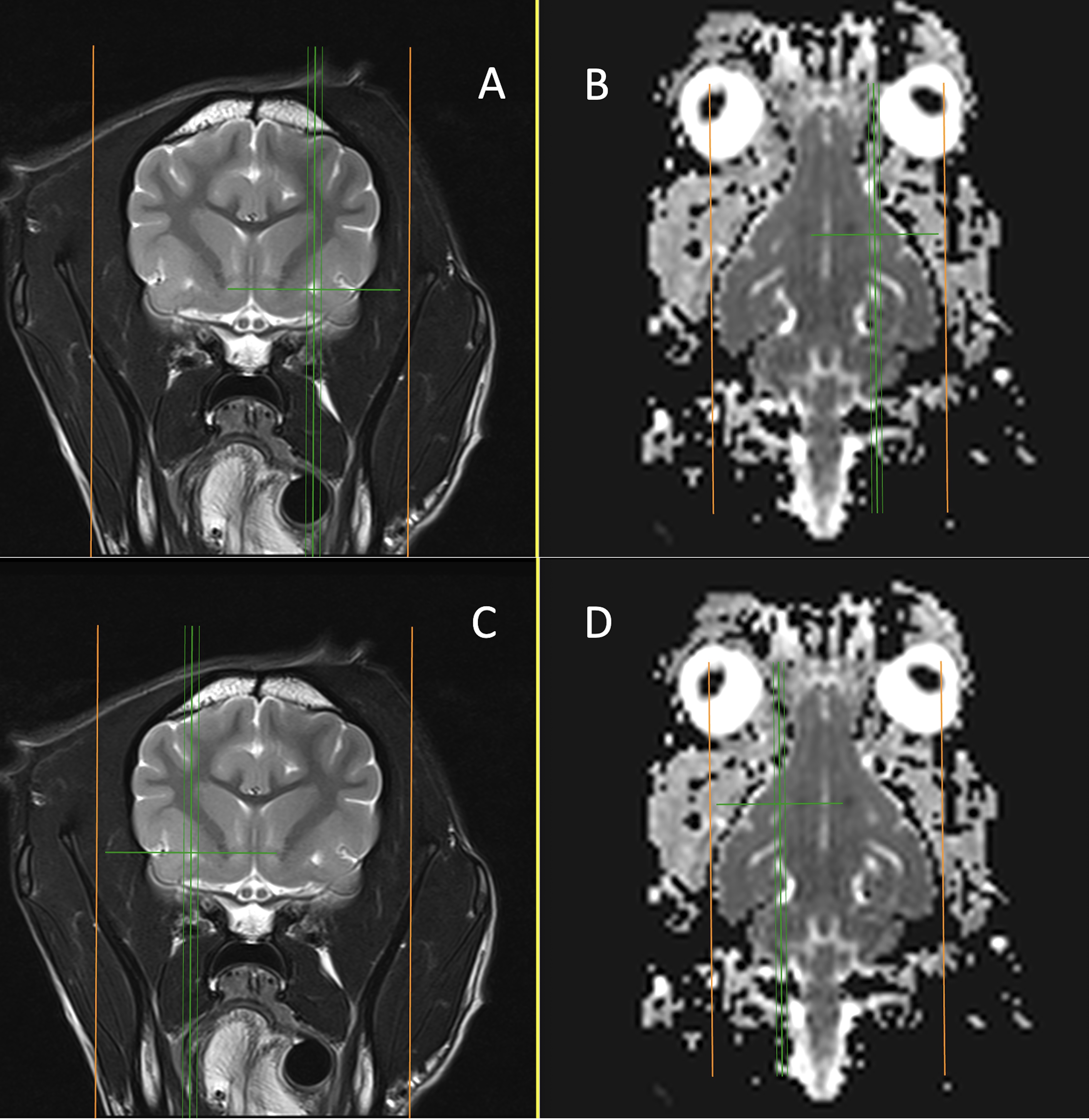

Supplement: Supplementary Figure 1 — (A,C) Transverse plane T2-W image of a canine brain with bilateral CSF-isointense foci in the ventral forebrain at the level of the rostral commissure. (B,D) corresponding dorsal plane Diffusion-Weighted ADC map showing no sign of restricted diffusion at the same location, indicated by the crosshairs. [file Image_1.JPEG]
